# Cecal volvulus mimicking acute appendicitis: A rare case report

**DOI:** 10.1016/j.ijscr.2025.110843

**Published:** 2025-01-04

**Authors:** Kalkidan Kibret Mekoya, Kaleab Habtemichael Gebreselassie, Tibebu Geremew Haile, Temesgen Mamo Bisetegn, Wegahtay Gebrekidan Tewelde

**Affiliations:** a Department of Surgery, Werabe University and Werabe Comprehensive Specialized Hospital, Werabe, Ethiopia; b Department of Surgery, Urology Unit, Werabe Comprehensive Specialized Hospital, Werabe, Ethiopia; c Department of Emergency and Critical care Medicine, Werabe Comprehensive Specialized Hospital, Werabe, Ethiopia; d Department of Surgery, Werabe Comprehensive Specialized Hospital, Werabe, Ethiopia; e Department of Surgery, Aksum University and Aksum Comprehensive Specialized Hospital, Aksum, Ethiopia

**Keywords:** Cecal volvulus, Defective right colon fixation

## Abstract

**Introduction:**

Cecal volvulus is a rare condition, accounting for about 1–5 % of intestinal obstruction causes. It carries high morbidity and mortality unless diagnosed and managed early.

**Case presentation:**

We present a case of 33 yrs. old male patient who initially presented with typical sign and symptoms of appendicitis but intraoperatively was found to have viable cecal volvulus.

**Discussion:**

Diagnosing cecal volvulus can be challenging because its presentation often mimics that of other causes of intestinal obstruction. Additional investigative modalities such as barium studies and CT scan are helpful in diagnosing the condition.

**Conclusion:**

Cecal volvulus is a clear emergency that require urgent surgery. In our case presentation highlight unusual clinical presentation of cecal volvulus.

## Highlights

• We present a case of cecal volvulus in 33 yrs. old male patient.
• Patient presentation was unusual, typical of Acute Appendicitis.
• Cecum and ascending colon lacks complete retroperitoneal fixation.
• De-torsion was done during the emergency procedure and Right hemi-colectomy and ileo-transverse anastomosis was done after 6 days.

## 1 Introduction

Cecal volvulus (CV) is an uncommon cause of acute intestinal obstruction, less common than sigmoid volvulus [1]. Predisposing anatomical characteristics include incomplete intestinal rotation during embryogenesis and insufficient retroperitoneal attachments of the right colon, either congenitally or as a result of mobilization during prior surgery [2]. Defective peritoneal fixation of the ascending colon and cecum occurs in approximately 10 % of the population and requires surgical intervention with serious morbidity if the diagnosis is delayed. For this reason, a high index of suspicion is necessary for diagnosis [3]. Early diagnosis is essential to reduce the high mortality rate reported with this condition, which is essentially a closed-loop obstruction that can lead to vascular compromise, gangrene, and perforation [1]. We present a rare case of cecal volvulus secondary to defective fixation of the ascending colon and cecum, with reporting following the SCARE guidelines [4].

## 2 Case presentation

A 33-year-old male patient presented with shifting right lower quadrant abdominal pain of 1 day's duration. He also had associated nausea, one episode of vomiting, and intermittent abdominal pain and constipation for the past two months. He reported no issues with passing feces or flatus, no abdominal distension, and no prior history of surgery. On physical examination, he had borderline tachycardia with a pulse rate of 98 beats per minute, normal temperature, direct and rebound tenderness in the right lower quadrant of the abdomen, tympanic to percussion, and no abdominal distension. He underwent a complete blood count (CBC) which showed normal results, with no significant inflammatory markers, ruling out severe infection or sepsis. Electrolytes and renal function were also normal, indicating no major fluid or electrolyte imbalance. An abdominopelvic ultrasound that showed simple intraperitoneal collection, dilated bowel loops, and a poorly visualized appendix. Other imaging investigations, such as abdominal X-ray and CT scan, were not performed because the clinical presentation was typical of acute appendicitis, and additional imaging is not routinely done in such cases in our setup.

**Fig. 1:**
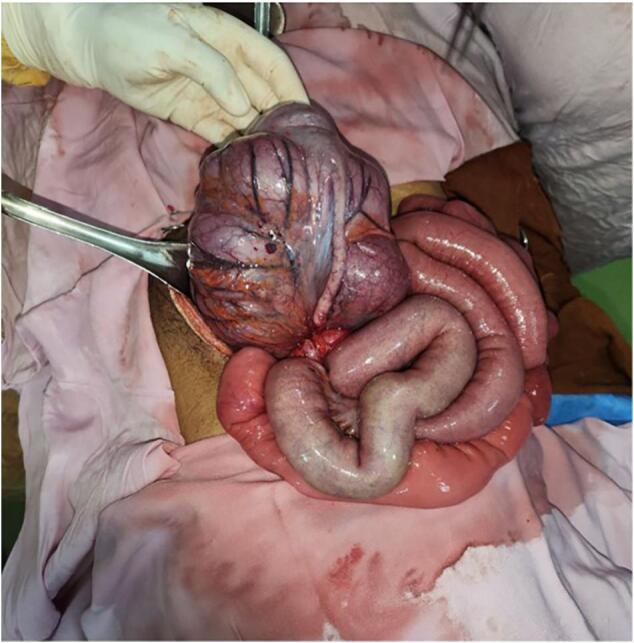
A, Intraoperative picture of axially volvulated cecum. In this image, the cecum is visibly twisted along its mesentery, creating an axial volvulus. The bowel appears markedly distended and dusky coloration.

**Fig. 2:**
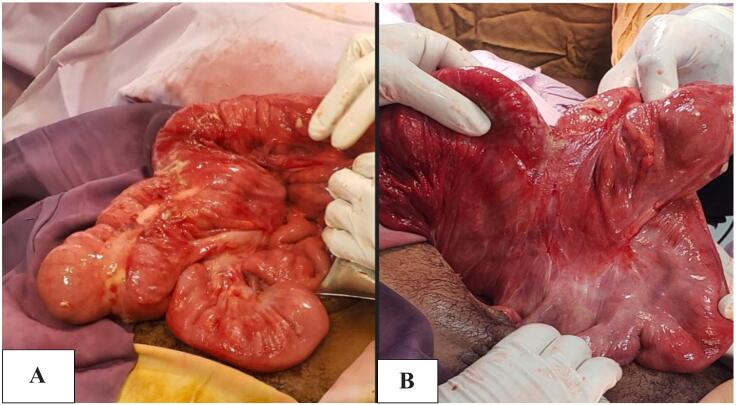
A, B Intraoperative picture of cecum and ascending colon. These images depict the cecum and ascending colon, which lack the usual retroperitoneal fixation. The cecum is shown to be mobile and freely hanging, which is a contributing factor to the volvulus.

With the typical symptom of shifting abdominal pain and direct and rebound right lower quadrant tenderness clinical diagnosis of acute appendicitis was settled and informed written consent was obtained. Patient was initially operated on with a right lower quadrant incision but finding of a volvulated cecum required changing the incision to a vertical midline approach. The cecum was dusky and twisted 270° along its mesentery (Fig. 1A). The entire right colon was mobile and lacked retroperitoneal fixation (Fig. 2A, B). After de-torsion and packing, the cecum regained its normal color.

Definitive management (right hemicolectomy) was deferred because the colon was heavily loaded with feces, which posed a significant risk of contamination and surgical complications. Immediate surgery could have led to difficulties in achieving a clean surgical field and increased the risk of perforation. Thus, the decision was made to stabilize the patient first with de-torsion and bowel preparation. After these measures, definitive surgery was performed 6 days later, reducing the risk of complications. The patient was followed up until the third postoperative month, during which he had a smooth recovery. He has been discharged from follow-up by the time of submitting this case report.

## 3 Discussion

Cecal volvulus was first described by Rokitansky in 1837 [6] and is a rare condition with an estimated incidence of 2.8–7.1 cases per million people per year (pediatric incidence is undefined), accounting for 1 %–5 % of all adult intestinal obstructions and 40 % of colonic volvuli [5]. There are two variants of cecal volvulus. The first and most common is an axial twist over the ileocolic vessels with early ischemic changes and gangrene. The second variant, known as the cecal bascule, involves only the anterior folding of the cecum to the ascending colon, and it rarely results in vascular compromise [7]. Identified risk factors for cecal volvulus include chronic constipation, high-fiber diet, pregnancy, abdominal mass, and being bedridden. There is conflicting evidence regarding the role of previous abdominal surgery as a risk factor. However, a hypermobile cecum is a key factor in the development of cecal volvulus [8]. Clinical presentations of cecal volvulus may include severe abdominal pain, constipation, abdominal distension and vomiting. Diagnosing cecal volvulus can be challenging, as its presentation often closely resembles that of other causes of intestinal obstruction [9].

Imaging techniques commonly employed in the diagnosis of cecal volvulus include plain abdominal radiography, barium enema, abdominal computed tomography scan and colonoscopy. Up to 30 % of patients do not show radiographic peculiarities, making diagnosis difficult and often delayed [10]. Additionally, the use of endoscopy in the diagnosis of acute cecal volvulus is limited because the success rate of colonoscopic reduction is only about 30 % [11]. Barium enema is 88 % accurate for detecting volvulus and allows visualization of the distal colon to exclude contributory abnormalities; it occasionally succeeds in reducing the volvulus [6]. On CT scan, three pathognomonic signs associated with acute cecal volvulus are: “coffee bean”, “bird beak”, and “whirl signs”. In the setting of acute cecal volvulus, the whirl sign consists of a spiraled loop of collapsed cecum, with low-attenuating fatty mesentery and engorged mesenteric vessels. Visualization of a gas-filled appendix is also a CT scan sign associated with cecal volvulus [12].

The recommended surgical procedure for cecal volvulus remains controversial, with the choice of procedure dependent on the clinical presentation of the patient [13]. Strategies for the management of cecal volvulus include surgical resection, endoscopic reduction, and cecopexy. Endoscopic reduction carries a significant risk of recurrence, whereas cecopexy is only suitable for volvulus with a viable viscus [14]. Resection, either limited or right hemicolectomy, is mandatory for gangrenous and a grossly distended, thin-walled cecum. Simple de-torsion, rectopexy, and cecostomy appear to be less effective and more morbid options than resection and anastomosis, even for viable bowel [15]. For de-torsion and cecopexy, the recurrence rate was between 20 % and 30 % [13]. Right hemicolectomy is the surgery of choice whenever gangrenous cecal volvulus is diagnosed, with a mortality rate comparable to that of other non-resectional methods, but a significantly low or zero recurrence rates [7]. Perioperative mortality for cecal volvulus is approximately 0–40 %, depending on the bowel viability, as well as the type of the therapeutic procedure. Early diagnosis is essential to reduce the high mortality rate [16].

## 4 Conclusion

In conclusion, our patient presented with a rare case of cecal volvulus characterized by the absence of fixation of the cecum and ascending colon. Notably, the clinical presentation closely mimicked that of acute appendicitis, which made the diagnosis challenging. During the emergency procedure, only de-torsion was performed, followed by bowel preparation and subsequent definitive surgery on an elective basis to mitigate the risk of recurrence. Although this approach carries the potential risk of re-volvulation in the postoperative period and reoperation, it successfully managed the patient's condition.

## Author contribution

Kalkidan Kibret Mekoya - Literature review, introduction, case presentation writing, compiling and writing the first draft.

Wegahtay Gebrekidan Tewelde - Literature review and writing the discussion and conclusion part.

Tibebu Geremew Haile - Lierature review and writing discussion and conclusion part.

Temesgen Mamo Bisetegn - Assisted in compiling and writing the first draft.

Kaleab Habtemichael Gebreselassie - Literature review, reviewed the first draft and writing the final draft.

## Consent

Written informed consent was obtained from the patient for publication and any accompanying images. A copy of the written consent is available for review by the Editor-in-Chief of this journal on request.

## Ethical approval

The case report is exempt from ethical approval. Ethical clearance is not necessary. The IRB of Werabe Comprehensive specialised Hospital has exempted case reports from requiring ethical clearance.

## Guarantor

Kalkidan Kibret.

## Research registration number

Research registration not needed.

## Funding

No funding was received.

## Conflict of interest statement

The authors declare no conflict of interest.
